# Systemic Inflammatory Parameters in Patients with Elderly-Onset Rheumatoid Arthritis (EORA) and Young-Onset Rheumatoid Arthritis (YORA)—An Observational Study

**DOI:** 10.3390/jcm10061204

**Published:** 2021-03-14

**Authors:** Bożena Targońska-Stępniak, Krzysztof Grzechnik, Katarzyna Kolarz, Danuta Gągoł, Maria Majdan

**Affiliations:** 1 Department of Rheumatology and Connective Tissue Diseases, Medical University of Lublin, 20-954 Lublin, Poland; maria.majdan@gmail.com; 2 Department of Rheumatology and Connective Tissue Diseases, Independent Public Teaching Hospital No 4, 20-954 Lublin, Poland; krzysiek.grzechnik@gmail.com (K.G.); katarzyna.kolarz@gmail.com (K.K.); d_rola@wp.pl (D.G.)

**Keywords:** rheumatoid arthritis, EORA, YORA, disease activity, systemic inflammatory markers, NLR, PLR

## Abstract

Background: Rheumatoid arthritis (RA) occurs more often in elderly individuals. Elderly onset RA (EORA) (onset > 60 years) encompasses a specific subset of patients if compared with young onset RA (YORA) (onset at a younger age). There is a need to define reliable, simple markers to properly assess the inflammatory activity of RA. Hematological markers of systemic inflammation (Platelet-To-Lymphocyte (PLR) and Neutrophil-To-Lymphocyte (NLR) ratios) are novel measures of the inflammatory response. The goal of the study was to analyze the course of EORA vs. YORA patients and to assess associations between systemic and clinical disease activity markers, including PLR and NLR, in different subsets of patients. PLR and NLR have not previously been assessed in EORA and YORA. Methods: The study group consisted of 113 consecutive patients (63 EORA and 50 YORA). The following assessments were performed: joint counts, Disease Activity Score (DAS28), complete blood cell counts, erythrocyte sedimentation rate (ESR), and C-reactive protein (CRP). Results: EORA was characterized by significantly higher disease activity markers (conventional inflammatory and clinical), a lower rate of remission or low disease activity, and less frequent use of biological drugs and glucocorticoids. The NLR and PLR were positively correlated with disease activity markers. The PLR was significantly lower in EORA compared with in YORA. Conclusion: EORA and YORA patients differed significantly. In EORA, conventional disease activity markers were higher, the PLR was significantly lower.

## 1. Introduction

Rheumatoid arthritis (RA) is a chronic, autoimmune disease, characterized by progressive, symmetric polyarthritis, leading to irreversible destruction and deformities of the joints. The disease affects primarily women and is characterized by an average age onset of 55 years, with a peak incidence between 30 and 50 years of age [1,2].

In the general population, life expectancy is increasing in recent decades. At the same time, the proportion of elderly patients with RA is increasing, and the incidence of RA occurs more often in elderly individuals. Elderly onset rheumatoid arthritis (EORA) is usually defined as the disease onset after 60 years of age. Researchers estimated that EORA contributes 10%–33% of all RA cases [3]. The term “young onset rheumatoid arthritis” (YORA) refers to the disease with a more typical onset at a younger age.

Research reported that EORA encompasses a specific subset of patients with RA. As compared with YORA, EORA is characterized by a more equal distribution of sex, lower incidence of rheumatoid factor (RF), higher frequency of acute onset with constitutional symptoms and large joint involvement [3,4], higher disease activity, and greater disability of patients [5,6]. Disease modifying anti-rheumatic drugs (DMARDs) are less often used in EORA than YORA patients, which may contribute to disability early in the disease course [5,6].

There is an ongoing need to define reliable, simple markers that could facilitate the proper assessment of RA inflammatory activity, supplementary to conventional inflammatory markers (erythrocyte sedimentation rate (ESR) and C-reactive protein (CRP). In the last few years, hematological parameters of systemic inflammation (the Neutrophil-to-Lymphocyte ratio (NLR) and Platelet-to-Lymphocyte ratio (PLR) have come into use. They have been reported to be sensitive measures of inflammation in different fields of medicine: oncology, diabetes, cardiology, nephrology, infectious diseases, and autoimmune rheumatic diseases, including RA [7,8,9,10,11]. Research found that NLR and PLR correlate with different parameters of RA activity (laboratory, clinical, and ultrasound) and may serve as reliable markers of RA systemic activity. However, NLR and PLR have never been assessed in specific subsets of RA patients (EORA and YORA).

The purpose of this study was to assess the value of hematological markers of systemic inflammation (NLR and PLR) in the assessment of disease activity, in different subsets of RA patients (EORA and YORA). An additional goal of the study was to analyze the course of the disease in EORA and YORA patients.

## 2. Materials and Methods

### 2.1. Study Population

The study group consisted of 113 RA patients, hospitalized in the Department of Rheumatology and Connective Tissue Diseases, Medical University of Lublin. All the patients fulfilled the American College of Rheumatology (ACR)/European League Against Rheumatism (EULAR) classification criteria for RA [12]. Patients were classified into two groups, according to the age of RA onset: EORA with the disease onset > 60 years of age and YORA with the disease onset between 18 and 30 years of age. A detailed comparative analysis was performed between the two groups of patients.

This study was conducted in accordance with the Declaration of Helsinki of 1975, revised in 2013. The design of the study was approved by the Ethics Committee of the Medical University of Lublin (approval number KE-0254/319/2016, obtained before undertaking the research). Informed consent was obtained from each patient, after an adequate explanation of the study design, prior to their inclusion in the study.

### 2.2. Clinical and Laboratory Findings

Baseline demographics and clinical data were collected through medical interviews and a review of their medical history and records. Data, including the age of the disease onset, RA duration, RF-IgM (IgM rheumatoid factor) and anti-citrullinated peptide antibodies (anti-CCP) serology, extra-articular manifestations, and comorbidities, were retrieved. Details on current and past therapy were also collected. The height and weight were measured barefoot wearing light clothes. The body mass index (BMI) was calculated as the ratio of weight to squared height.

A physical examination was performed, including the tender joint count (TJC) and swollen joint count (SJC). The disease activity of RA was determined using the 28 joints Disease Activity Score system (DAS28), calculated with the TJC, SJC, ESR, and patient global assessment (PGA) in a visual analogue scale (VAS) [13]. The cut point for remission was a DAS28 value ≤ 2.6 and low disease activity (LDA) ≤ 3.2.

The erosive form of RA was identified in patients who had erosions on joint surfaces of bones in X-rays of hands and/or feet, as assessed according to the Sharp/van der Heijde score by a trained radiologist [14].

Blood was collected after overnight fasting. Blood tests in all patients included a complete blood cell count (CBC), ESR, CRP, serum creatinine, and uric acid. The NLR was calculated by dividing the absolute neutrophil count (ANC) by the absolute lymphocyte count (ALC). The PLR was calculated by dividing the absolute platelet (PLT) count by the ALC.

### 2.3. Statistical Analysis

Continuous variables are presented using the mean ± standard deviation (SD) or median and interquartile range (IQR) if the data were parametric or nonparametric, respectively. Categorical data were summarized as absolute numbers and percentages. The results were tested for normality using the Kolmogorov–Smirnov’s test. To compare continuous variables in subgroups of patients, Student’s *t*-test or the nonparametric Mann–Whitney *U* test, were used. Correlation between the quantitative variables was assessed by Spearman’s or Pearson’s correlation test. Categorical data were compared using the chi-square test. Multiple linear regression test was performed introducing variables that showed statistically significant association with certain parameters. For all tests, *p* values < 0.05 were considered significant. All statistical analyses were performed using the StatSoft STATISTICA 12 application.

## 3. Results

### 3.1. Demographic and Disease-Related Variables in 113 RA Patients

The study group consisted mostly of women (female vs. male: 93 (82.3%) vs. 20 (17.7%)). The disease duration was 12.9 (±10.3) years. The vast majority of patients had an erosive form of RA (105 (92.9%)) and were seropositive for RF-IgM and/or anti-CCP (108 (95.6%)). Extra-articular symptoms in the course of the disease were observed in 33 (29.2%) patients (sicca syndrome in 21 patients, interstitial lung disease in 7 patients, rheumatoid nodules in 5 patients, and amyloidosis in 1 patient).

Remission or LDA at the time of examination was found in 40 (35.4%) patients. Conventional synthetic DMARDs (csDMARDs) were used in all patients and included (in monotherapy or combination): methotrexate (MTX) in 68 (60.2%) patients (dose 10–25 mg/week), leflunomide (LEF) in 23 (20.4%), hydroxychloroquine (HCQ) or chloroquine (CQ) in 28 (24.8%), and sulfasalazine (SS) in 10 (8.8%). Low-dose glucocorticoid (GC) therapy (prednisone ≤ 7.5 mg/day) was used in 96 (85.0%) patients. Biological DMARDs were used in 28 (24.8%) of patients.

### 3.2. Clinical Characteristics of Patients with YORA and EORA

Patients with EORA as compared to YORA, were characterized by significantly higher age and shorter disease duration. Significantly more men constituted the group of EORA compared to YORA (16 (25.4%) vs. 4 (8%)) (Table 1).

Joint involvement at the disease onset did not differ between the groups. We found that the small joints of the hands, wrists, and knees were involved the most commonly in both groups, as well as the small joints of the feet in YORA and shoulders in EORA. The vast majority of EORA and YORA patients were seropositive, either for RF or ACPA, or both antibodies. Only a few patients were seronegative in both groups (Table 1).

The incidence of extra-articular manifestations in the course of the disease was similar in both groups (Table 1). The following extra-articular manifestations were observed, in EORA patients: sicca syndrome in 10, interstitial lung disease in 5 cases; and in YORA patients: sicca syndrome in 10, rheumatoid nodules in 4, interstitial lung disease in 2 cases, amyloidosis in 1, and both sicca syndrome and rheumatoid nodules in 1 patient. Erosions of the hands and/or feet were found in over 90% of patients in both groups (Table 1).

Concomitant diseases were noted significantly more often in EORA than YORA patients (Table 1). The following concomitant diseases were observed, in EORA patients: arterial hypertension in 34, autoimmune thyroid disease in 17, diabetes in 8, ischemic heart disease in 10, metabolic syndrome in 8, cholelithiasis in 4, heart failure in 4 patients; and in YORA patients: arterial hypertension in 15, autoimmune thyroid disease in 10, diabetes in 3, ischemic heart disease in 1, metabolic syndrome in 1, cholelithiasis in 2 cases. At the time of examination concomitant diseases were stable and did not affect the inflammatory activity of RA. The metabolic parameters were significantly higher in EORA patients (BMI, serum concentration of creatinine, and uric acid) (Table 1).

We found that non-steroidal anti-inflammatory drugs (NSAIDs) were used significantly more often in EORA patients, as the first treatment choice. The NSAID was chosen as the first drug in EORA at the disease onset, and it was replaced by DMARD. The therapy with NSAID was of short duration, there were no significant adverse events associated with NSAIDs (Table 1).

MTX was used as the first DMARD more often in EORA than in YORA patients. Concurrently, SS and other drugs (gold salts) were used as the first DMARD significantly more often in YORA than in EORA patients (Table 1).

The current GC treatment was used more often in YORA patients. It was low dose therapy (prednisone ≤ 7.5 mg/day), mostly of chronic nature (Table 1).

Biological DMARDs were used significantly less often in EORA than in YORA patients, in respect to both tumor necrosis factor inhibitors (TNFi) and other biological drugs (Table 1).

### 3.3. Disease Activity Parameters in Patients with YORA and EORA

**Figure 1:**
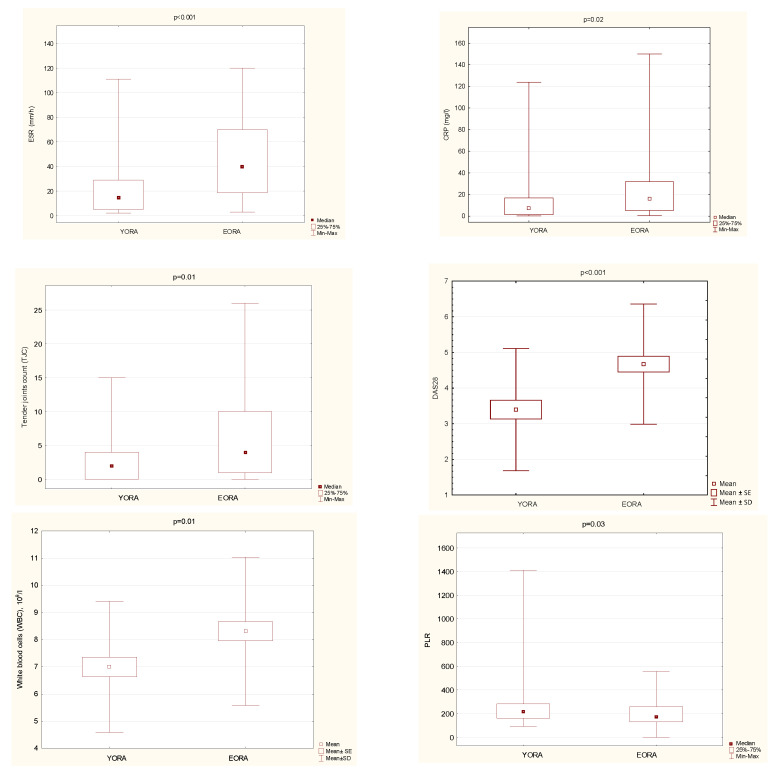
Significant differences between patients with YORA vs. EORA.

Patients with EORA, compared with YORA, were characterized by significantly higher values of several disease activity markers: both clinical (DAS28 and TJC) and systemic inflammatory parameters (ESR and CRP), as well as higher white blood cell counts (WBC) and ANC (Table 2) (Figure 1). The value of PLT, ALC, and the hemoglobin concentration did not differ significantly between the groups. (Table 2).

The value of PLR was significantly lower in EORA compared with YORA patients (Figure 1). The NLR value was comparable in both groups (Table 2).

Remission or LDA was achieved significantly less often in EORA compared to YORA patients (Table 2).

### 3.4. Relationship between the Disease Activity and Metabolic Parameters with Timewise and Hematological Inflammatory Markers (NLR and PLR) in 113 RA Patients

In the whole group of RA patients, significant positive correlations were found between the age of RA onset and disease activity markers (CRP, ESR, DAS28, PGA, and ANC) as well as metabolic parameters (creatinine, uric acid, and BMI). Significant, positive associations were also noticed between the current patient age and disease activity markers (CRP, ESR, DAS28, PGA, and TJC) and metabolic parameters (creatinine, uric acid, and BMI) (Table 3). The disease duration was positively correlated with the WBC and ANC (Table 3).

A significant, negative correlation was noted between the age at RA onset and PLR value (Table 3). The NLR and PLR were significantly, positively associated with all the disease activity parameters, both laboratory (CRP and ESR) and clinical (DAS28, PGA, TJC, and SJC), as well as with PLT, ANC, and WBC (only NLR). There were also negative correlations between the NLR and PLR with the ALC, as well as with hemoglobin (only PLR) (Table 3).

### 3.5. Relationship between Hematological Systemic Inflammatory Markers (NLR and PLR) and Disease Activity Parameters in EORA and YORA Patients

In both groups, the NLR and PLR were significantly, positively associated with laboratory inflammatory parameters (CRP and ESR), PLT, ANC, and clinical markers of the disease activity (DAS28, TJC, and PGA). There was a negative association with the ALC (Table 4).

Additionally, the EORA group was characterized by positive associations between both the NLR and PLR with the SJC and duration of morning stiffness (Table 4). Concurrently, the YORA group was characterized by a negative correlation between the PLR and hemoglobin (Table 4).

In the multiple linear regression analysis, significant positive associations were confirmed in EORA patients for PLR with PLT (b = 0.57, *p* < 0.001) and PLR with ANC (b = 0.16, *p* < 0.03) and negative for PLR with ALC (b = −0.54, *p* < 0.001), as well as positive for NLR with ANC (b = 0.93, *p* < 0.001) and NLR with TJC (b = 0.17, *p* = 0.04).

In the multiple linear regression analysis, a significant positive association was confirmed in YORA patients for PLR with PLT (b = 0.5, *p* = 0.002) and a negative association for PLR with ALC (b = −0.82, *p* < 0.001), as well as a negative association for NLR with ALC (b = −0.67, *p* = 0.01).

## 4. Discussion

In our study, the value of the PLR was significantly lower in EORA compared with in YORA patients. We found that the age at RA onset was negatively correlated with the PLR. Simultaneously, the PLT count and ALC were comparable in EORA and YORA patients, with a tendency toward a lower PLT count and higher ALC in the EORA group (statistically not significant).

The hematological markers of systemic inflammation (NLR and PLR) were significantly, positively correlated with the disease activity markers, both laboratory (CRP and ESR) and clinical (DAS28, PGA, TJC, and SJC).

We observed significant, positive associations between both the age at RA onset and the patients’ current age with conventional disease activity markers: laboratory (CRP and ESR) and clinical (DAS28, PGA, and TJC), as well as with metabolic parameters. These associations did not occur in relation to the disease duration.

The results of the study showed that the EORA patients differed significantly from the YORA patients. The group of EORA was characterized by significantly higher disease activity markers, higher conventional inflammatory (CRP and ESR) and clinical (DAS28 and TJC) parameters. The target of RA treatment (remission or LDA) was achieved less often in patients with EORA. The method of RA treatment was also different. In patients with EORA, NSAID was the first drug used at the disease onset (~60% patients) and MTX was used as the first DMARD (~85% patients). Simultaneously, EORA patients were treated less frequently with biological DMARDs and GC. These differences might be associated with the significantly older age of EORA patients, more common comorbidities, and unfavorable metabolic parameters, which affect the choice of treatment.

According to the EULAR recommendations, GC treatment should be associated with screening for comorbidity and monitoring for possible adverse events (diabetes, hypertension, weight gain, infections, osteoporotic fractures, osteonecrosis, myopathy, eye problems, skin problems and neuropsychological adverse events) [15]. NSAIDs in the elderly should be prescribed with caution. NSAIDs cause inhibition of prostaglandin and thromboxane synthesis leading to renal vasoconstriction and reduced renal perfusion. Elderly patients are at higher risk of developing nephrotoxicity from NSAIDs. It is also known that gastro-intestinal bleeding and ulceration from NSAIDs use increase in severity and frequency with increasing age. All NSAIDs may be associated with increased cardiovascular adverse effects, especially NSAIDs can increase blood pressure. Moreover, NSAIDs are one of the most common causes of adverse drug reactions, which is important if the number of medications increase [16].

Our results are consistent with the data in the literature. Research reported that, in EORA, disease activity is typically more marked, with higher SJC and TJC, longer duration of morning stiffness [3]. In EORA patients, higher values of DAS28 and SJC, acute phase reactants (CRP and ESR) and a comorbidity burden were observed [4,5]. Greater disease activity in EORA patients may contribute to disability early in the disease course [5]. Studies reported that EORA patients received more initial DMARD monotherapy and GC, but less combination DMARD with biological drugs, in comparison with YORA [5,6]. Improvement in the disease activity and physical function was significantly less in elderly patients. Treatment with TNFi significantly reduced the disease activity; however, it appeared less effective in elderly compared with younger RA patients. Good response or remission was achieved less often in older patients [17].

The results of our research may be associated with the previously reported PLT count decrease, which was observed in aged individuals, from various ethnic populations across the world [18,19]. Research reported that PLT counts remained stable in young to middle-aged subjects (20–60 years old) but dropped by approximately 10% in older individuals (>70 year of age) [18]. The mechanism resulting in the decreasing PLT count in older age remains unknown. Researchers suggested that a reduction in the hematopoietic stem cell reserve in older patients could be responsible for the reduced PLT production.

Another explanation is a shift in the hematopoietic stem cell population toward a megakaryocytic bias [18,20]. Although PLT counts decrease with aging, the PLT reactivity generally increased, leading to increased interactions with other cells and exaggerated inflammation. PLT signaling to monocytes in aging results in significantly more cytokine production. Increased PLT hyperactivity was observed in diseases associated with aging, such as sepsis, cardiovascular diseases, exaggerated inflammation, and thrombosis [18].

The PLR value reflects variations in the PLT and ALC. The PLR is more stable than the ALC or PLT count, as several physiological and pathological conditions could alter these cell counts. PLR is regarded as a biomarker for evaluating subclinical inflammation and an important indicator of systemic inflammation in non-oncologic diseases, such as cardiovascular disease (CVD), diabetes, sarcopenia, and autoimmune diseases [21,22]. A higher PLR is also associated with poor clinical outcomes in CVD and a poor prognosis in various oncologic diseases [22,23]. In a study including laryngeal cancer patients, PLR was categorized as low (≤119.55), moderate (>119.55 and ≤193.55), and high (>193.55). Only patients with high PLR experienced poor outcomes, including malnutrition and a more advanced cancer stage [24].

NLR is reported as an effective marker in inflammatory disorders of predictive value of poor outcomes in oncology and inflammatory disorders [25]. In patients with active RA, the ANC is increased due to increased anti-apoptotic cytokines and stimulation of myeloid cells, as well as by the granulocyte colony-stimulating factor. Lymphopenia in peripheral blood might be the result of the persistent accumulation of lymphocytes at the sites of inflammatory joints and increased apoptosis of lymphocytes in RA [11]. Lymphopenia is reported to be associated with decreased peripheral T cell counts due to their migration to the synovium [26]. Peripheral lymphopenia and the gradual increase in the neutrophil count have been often noted with the progression of RA. The NLR cut-off value of 1.4 classified patients in deep remission with 90% specificity and 24% sensitivity [27].

Another hematological parameter, red blood cell distribution width (RDW), is reported to be an inflammatory marker, positively correlated with conventional inflammatory markers (CRP, ESR), in unselected outpatients and in healthy population [28]. Other studies found close association of RDW with onset, activity and prognosis of inflammatory and autoimmune diseases such as RA, systemic lupus erythematosus, Sjögren syndrome, inflammatory bowel disease [29]. RDW appears to be insensitive to infective events and seems to represent long-term inflammatory status, while conventional parameters (CRP, ESR) represent short-term inflammatory status [26].

Our research demonstrated higher WBC and ANC in EORA, but no lymphopenia and no high PLT count, resulting in significantly lower PLR and comparable NLR values in EORA compared to YORA patients. The lower PLR value may result from the restricted involvement of PLT and ALC in the inflammatory status and decreased shift toward local inflammatory sites (joints) in EORA patients, and may point to better prognosis. Higher values of conventional inflammatory parameters (CRP and ESR) in EORA may be associated with comorbidities, which occur in aging. The findings of our research suggest that PLR might be of prognostic value in patients with EORA, similar to patients with other diseases. The relevance of PLR and NLR in EORA requires further prospective observation.

Our study has certain potential limitations. First is the relatively small number of patients included in the study; a higher number of patients could enable better statistical evaluation. Second, we presented data of a single clinical assessment of EORA and YORA patients; prospective research could be important. Third, an assessment of other, non-traditional parameters of inflammatory response (e.g., serum amyloid A (SAA)) or hematological markers of inflammatory process (e.g., RDW) could be valuable.

The study also has several strengths. First, to our best knowledge, this is the first study assessing the hematological parameters of systemic inflammation (NLR and PLR) in patients with different ages of RA onset (EORA and YORA). Second, the detailed clinical characteristics of the patients is presented, considering all aspects of RA pathology. Third, patients were not selected for the study, it was a real world study. Fourth, all assessments performed are quite inexpensive and available on an outpatient basis.

## 5. Conclusions

The results of our study showed significant differences between the compared groups of patients, with EORA and YORA. The EORA group consisted of significantly more men and was characterized by higher conventional parameters of disease activity [clinical (DAS28 and TJC) and laboratory (CRP and ESR)], less frequent remission or LDA, and more common comorbidities. The PLR was significantly lower in EORA in reference to the YORA group.

## Figures and Tables

**Table 1:** Comparison of clinical data in patients with and young-onset rheumatoid arthritis (YORA) and elderly-onset rheumatoid arthritis (EORA).

Data	YORA (*n* = 50)	EORA (*n* = 63)	*p*-Value
Age, years	41.5 (±13.7)	73.6 (±6.6)	<0.001
Gender, female/male (*n*,%)	46 (92.0)/ 4 (8.0)	47 (74.6)/ 16 (25.4)	0.02
Body mass index (BMI), kg/m^2^	23.4 (±4.1)	26.0 (±5.8)	0.04
Concomitant diseases (*n*,%)	40 (80.0)	61 (96.8)	0.004
**Rheumatoid Arthritis (RA)-Related Variables:**			
Disease duration, years	18.3 (±12.3)	8.6 (±5.4)	<0.001
Age at RA diagnosis, years	24 (20–27)	64 (62–67)	<0.001
Joint involvement at RA onset (*n*,%)			
small hand joints	43 (86)	54 (85.7)	
wrist	36 (72)	44 (69.8)	
elbow	18 (36)	16 (25.4)	NS
shoulder	19 (38)	29 (46)	
small joints of feet	21 (42)	18 (28.6)	
ankle	14 (28)	13 (20.6)	
knee	31 (62)	40 (63.5)	
hip	2 (4)	8 (12.7)	
Positive RF-IgM (*n*,%)	42 (84.0)	57 (90.5)	NS
Positive anti-CCP (*n*,%)	43 (86.0)	57 (90.5)	NS
Positive both (RF-IgM, anti-CCP) (*n*,%)	39 (78.0)	54 (90.0)	NS
Seronegative (*n*,%)	4 (8.0)	1 (1.6)	NS
Extra-articular manifestations (*n*,%)	18 (36.0)	15 (23.8)	NS
Erosions (hands/feet) (*n*,%)	48 (96.0)	57 (90.5)	NS
First drug used (*n*,%)			
NSAID	20 (40.0)	38 (60.3)	0.03
GC	11 (22.0)	6 (9.5)	NS
DMARD	19 (38)	19 (30.2)	NS
First DMARD used (*n*,%)			
MTX	26 (52)	54 (85.7)	<0.001
Anti-malarial drug	6 (12)	6 (9.5)	NS
Sulphalazin	11 (22.0)	3 (4.8)	0.01
Other	7 (14.0)	0	0.002
Current biological treatment (*n*,%)	34 (68.0)	6 (9.5)	<0.001
Anti-TNF	23 (46.0)	5 (7.9)	<0.001
Other	11 (22.0)	1 (1.6)	<0.001
Current low dose glucocorticoid use (*n*,%)	47 (94.0)	49 (77.8)	0.02

Values are displayed as mean ± standard deviation (SD), median (IQR) or frequencies with the corresponding percentages (%). anti-CCP, anti-cyclic citrullinated peptide antibodies; DMARD, diseases modifying anti-rheumatic drug; GC, glucocorticosteroid; NSAID, non-steroidal anti-inflammatory drug; RA, rheumatoid arthritis; and RF-IgM, IgM rheumatoid factor.

**Table 2:** Laboratory and clinical parameters in RA patients.

Data	All RA patients (*n* = 113)	YORA (*n* = 50)	EORA (*n* = 63)	*p*-Value (YORA vs. EORA)
**Laboratory Results:**				
Hemoglobin, g/dL	12.5 (±1.3)	12.5 (±1.3)	12.4 (±1.3)	NS
PLT,10^9^/L	306.0 (±98.8)	310.3 (±86.5)	302.7 (±107.8)	NS
WBC, 10^9^/L	7.7 (±2.7)	7.0 (±2.4)	8.3 (±2.7)	0.01
ANC, 10^9^/L	5.7 (±3.3)	4.8 (±2.1)	6.3 (±3.8)	0.02
ALC, 10^9^/L	1.6 (±0.8)	1.5 (±0.6)	1.7 (±0.9)	NS
NLR	3.06 (2.4–5.2)	3.0 (2.1–4.8)	3.1 (2.3–5.6)	NS
PLR	187.9 (138.7–265.2)	216.8 (161.6–282.0)	169.1 (129.6–258.4)	0.03
CRP, mg/L	10.8 (2.8–29.7)	7.2 (1.3–16.8)	16.0 (5.3–32.0)	0.02
ESR, mm/h	26 (12–58)	15 (5–29)	39.5 (18.5–70)	<0.001
Creatinine, mg/dL	0.8 (0.6–0.9)	0.7 (0.6–0.8)	0.9 (0.7–1.0)	<0.001
Uric acid, mg/dL	5.3 (±1.6)	4.4 (±1.6)	5.7 (±1.5)	0.001
**Clinical Parameters of RA Activity:**				
TJC	3 (1–7)	2 (0–4)	4 (1–10)	0.01
SJC	1 (0–4)	1 (0–4)	2 (0–5)	NS
PGA (VAS), mm	34.9 (±25.6)	29.4 (±27.3)	39.1 (±23.6)	NS
Morning stiffness, minutes	75.0 (±57.6)	51.5 (±58.8)	83.5 (±55.8)	NS
DAS28	3.9 (±1.7)	3.2 (±1.6)	4.4 (±1.5)	<0.001
Remission or Low Disease Activity (DAS28 < 3.2) (*n*, %)	40 (35.4)	25 (50.0)	15 (23.8)	0.004

Values are displayed as mean ± standard deviation (SD), median (IQR) or frequencies with corresponding percentages (%) ALC, absolute lymphocyte count; ANC, absolute neutrophil count; CRP, C-reactive protein; DAS28, disease activity score in 28 joints; ESR, erythrocyte sedimentation rate; NLR, neutrophil-lymphocyte ratio; PGA, patient global assessment; PLR, platelet-lymphocyte ratio; PLT-platelet count; RA, rheumatoid arthritis; SJC, swollen joints count TJC, tender joint count; VAS, Visual Analogue Scale; and WBC, white blood cell count.

**Table 3:** Associations with timewise and hematological systemic inflammatory parameters in 113 RA patients.

Data/R Spearman	Age	Age at RA Onset	Disease Duration	NLR	PLR
CRP	0.31 ***	0.23 **	NS	0.58 ***	0.44 ***
ESR	0.4 ***	0.37 ***	NS	0.39 ***	0.36 ***
DAS28	0.39 ***	0.33 ***	NS	0.53 ***	0.3 ***
PGA (VAS)	0.32 ***	0.23 **	NS	0.46 ***	0.24 **
TJC	NS	NS	NS	0.39 ***	0.25 **
SJC	NS	NS	NS	0.4 ***	0.35 ***
PLR	NS	−0.2 *	NS	0.67 ***	-
NLR	NS	NS	NS	-	0.67 ***
WBC	NS	NS	0.25 **	0.45 ***	NS
ANC	NS	0.19 *	0.23 **	0.72 ***	0.22 **
ALC	NS	NS	Ns	−0.52 ***	−0.69 ***
PLT	NS	NS	NS	0.34 ***	0.54 ***
Hb	NS	NS	NS	NS	−0.24 **
Creatinine	0.37 ***	0.36 ***	NS	NS	NS
Uric acid	0.37 ***	0.35 ***	NS	NS	NS
BMI	0.25 **	0.27 **	NS	NS	NS

ALC, absolute lymphocyte count; ANC, absolute neutrophil count; BMI, body mass index; CRP, C-reactive protein; DAS28, disease activity score in 28 joints; ESR, erythrocyte sedimentation rate; NLR, neutrophil-lymphocyte ratio; PGA, patient global assessment; PLR, platelet-lymphocyte ratio; RA, rheumatoid arthritis; SJC, swollen joint count; TJC, tender joint count; VAS, Visual Analogue Scale; and WBC, white blood cell count. * *p* < 0.05; ** *p* < 0.02; and *** *p* < 0.01.

**Table 4:** Associations between hematological systemic inflammatory markers and disease activity parameters in YORA and EORA.

Data R Spearman	PLR		NLR	
	YORA	EORA	YORA	EORA
CRP	0.35 *	0.61 ***	0.5 ***	0.63 ***
ESR	0.41 ***	0.35 ***	0.35 **	0.44 ***
Hb	−0.3 *	NS	NS	NS
PLT	0.5 ***	0.52 ***	0.46 ***	0.31 **
WBC	NS	NS	0.42 ***	0.48 ***
ANC	0.21 **	0.25 ***	0.67 ***	0.76 ***
ALC	−0.81 ***	−0.61 ***	−0.52 ***	−0.57 ***
TJC	0.33 *	0.27 *	0.47 ***	0.34 ***
SJC	NS	0.45 ***	NS	0.49 ***
PGA (VAS)	0.24 ***	0.36 **	0.43 ***	0.43 ***
DAS28	0.42 ***	0.35 ***	0.57 ***	0.54 ***
Morning stiffness	NS	0.5 ***	NS	0.56 ***
NLR	0.7 ***	0.7 ***	-	-
PLR	-	-	0.7 ***	0.7 ***

ALC, absolute lymphocyte count; ANC, absolute neutrophil count; CRP, C-reactive protein; DAS28, disease activity score in 28 joints; ESR, erythrocyte sedimentation rate; Hb, hemoglobin; NLR, neutrophil-lymphocyte ratio; PGA, patient global assessment; PLT, platelet count; PLR, platelet-lymphocyte ratio; RA, rheumatoid arthritis; SJC, swollen joints count; TJC, tender joint count; VAS, Visual Analogue Scale; and WBC, white blood cell count. * *p* < 0.05; ** *p* < 0.02; and *** *p* < 0.01.

## Data Availability

The data presented in this study are available on request from the corresponding author. The data are not publicly available because they include patients’ personal data.
